# Novel echocardiographic parameters in pulmonary hypertension assessment and the relationship between echocardiography and 6-minute walking test

**DOI:** 10.1097/MD.0000000000035294

**Published:** 2024-02-09

**Authors:** Leila Bigdelu, Hedieh Alimi, Afsoon Fazlinezhad, Fereshteh Ghaderi, Hoorak Poorzand, Farveh Vakilian, Faeze Keihanian, Zahra Abbasi Shaye, Sara Afshar

**Affiliations:** a Faculty of Medicine, Department of Cardiology, Ghaem Hospital, Mashhad University of Medical Sciences, Mashhad, Iran; b Faculty of Medicine, Department of Cardiology, Imam Reza Hospital, Mashhad University of Medical Sciences, Mashhad, Iran; c Faculty of Medicine, Department of Cardiology, Imam Reza and Ghaem Hospital, Mashhad University of Medical Sciences, Mashhad, Iran; d Clinical Research Development Center, Faculty of Medicine, Akbar Hospital, Mashhad University of Medical Sciences, Mashhad, Iran.

**Keywords:** functional capacity, pulmonary artery hypertension, pulmonary artery stiffness

## Abstract

Pulmonary artery stiffness (PAS) has been shown to be related to pulmonary artery pressure in patients with pulmonary artery hypertension (PAH). The aim of this study was to determine the correlation between functional capacity and echocardiographic indices of PAS in patients with PAH. This cross-sectional study was performed on patients with PAH who were confirmed by right heart catheterization and referred to Imam Reza PAH clinic for routine follow-up between November 2019 and January 2020. All patients underwent echocardiography and the maximum Doppler frequency shift, pulmonary acceleration time, peak velocity of the pulmonary flow, and velocity time integral, as well as PAS, were measured. All patients performed a 6-minute walk test. Fifty patients with a mean age of 41.90 ± 14.73 years old participated in this study. The majority of the patients were female (74%). The most common cause of PAH was idiopathic (74%). There was a significant correlation between PAS and pulmonary artery systolic pressure (*r* = 0.302, *P* = .041), second pulmonary valve pulse Doppler velocity (V_2_) (*r* = −0.461, *P* = .003), time from onset of pulmonary flow ejection to V_2_/first pulmonary valve pulse Doppler velocity (*r* = −0.311, *P* = .037) and Z_3_ ratio (*r* = −0.346, *P* = .023). There was no significant correlation between PAS and 6-minute walk test, pulmonary vascular resistance, and tricuspid annular plane systolic excursion (*P* > .05). There was a significant correlation between V_2_ and pulmonary vascular resistance (*r* = 0.359, *P* = .049). PAS and first pulmonary valve pulse Doppler velocity are simple, noninvasive, available tools for the evaluation of pulmonary vascular beds and diagnosis of presymptomatic clinical status in patients with PAH.

## 1. Introduction

Pulmonary arterial hypertension (PAH) is defined as mean pulmonary arterial pressure (mPAP) ≥25 mm Hg at rest.^[1]^ PAH is a progressive disorder that primarily results from extensive pulmonary vascular remodeling over time.^[2]^ PAH is a complex disorder and can be the end result of a variety of different underlying disorders. Pulmonary artery stiffness (PAS) is a strong determinant of right ventricular (RV) functions.^[3]^ PAS is mainly due to endothelial dysfunction and inflammation.^[4]^ Elevated PAS leads to a higher right ventricle workload and enhances transmission to small pulmonary vessels, which results in further vascular damage.^[1,5]^ Increased stiffness is associated with reduced functional capacity^[6]^ and higher mortality.^[7]^ This association is independent of pulmonary artery pressures or pulmonary vascular resistance.^[7]^ So far, PAS and response to treatment have been assessed through invasive hemodynamic assessment.^[8–10]^ Noninvasive imaging modalities have also been used to measure PA distensibility for the assessment of pulmonary artery (PA) stiffness and risk stratification in patients with PAH.^[11]^ Many studies demonstrate that PAS is correlated with RV dysfunction and disease severity in PAH regardless of the etiology.^[12,13]^

Despite a good correlation between mPAP and pulmonary artery acceleration time, some errors exist in the noninvasive estimation of mPAP using pulmonary acceleration time by Doppler echocardiography.^[14]^ Studies found a correlation between pulmonary artery elasticity and PAP.^[15]^ This indicates that PAS parameters can be used to determine PAH and evaluate RV function.

In this study, we aimed to determine the correlation between the 6-minute walk test (6MWT), as an indicator of functional capacity, with echocardiographic indices of PAS in patients with PAH.

## 2. Methods

### 2.1. Subjects

This cross-sectional study was performed on patients referred to the Imam Reza PH clinic for routine follow-up (including 6MWT) between November 2019 and January 2020.

### 2.2. Eligibility

Patients with a confirmed diagnosis of PAH based on right heart catheterization within the past 12 months were enrolled in this study. PAH was defined as mPAP >25 mm Hg, pulmonary artery wedge pressure ≤15 mm Hg at rest. We included patients younger than 65 years old who were diagnosed with PAH within the past 12 months. Subjects with diabetes mellitus, chronic kidney disease, and peripheral vascular disease, or a history of stroke and significant anemia were excluded. We also excluded patients with moderate or severe mitral and aortic regurgitation or stenosis, tricuspid stenosis, previous aorta or mitral valve operation or valvuloplasty, clinical, electrocardiographic, or angiographic evidence for coronary artery disease; wall motion abnormality, and bundle branch block and arrhythmia.

### 2.3. Six-minute walk test

The 6MWT was carried out by a trained technician on a 20-m corridor with no prior practice walks. Patients were instructed to walk and cover as much distance as possible in 6 minutes. Standardized encouragement was given at 1-minute intervals as per guideline.^[16]^

### 2.4. Transthoracic echocardiography

Transthoracic echocardiography was performed by an experienced echocardiography specialist the day before or after 6MWT using the Philips Echocardiography system iE33 equipped with an S5 probe in the left lateral decubitus position. The recordings were performed in 5 cardiac cycles. Measurements were adjusted to obtain maximal frequency shifts. Doppler signals were recorded at the speed of 100 mm/s. The maximum Doppler frequency shift, pulmonary acceleration time, peak velocity of the pulmonary flow, and velocity time integral were measured from the parasternal short-axis view using pulsed Doppler ultrasound with the sample volume placed in the pulmonary artery just 1 cm below the pulmonary valve annulus. Pulmonary artery stiffness was calculated by dividing maximal frequency shift by pulmonary acceleration time.

### 2.5. Measurements

**Figure 1.**
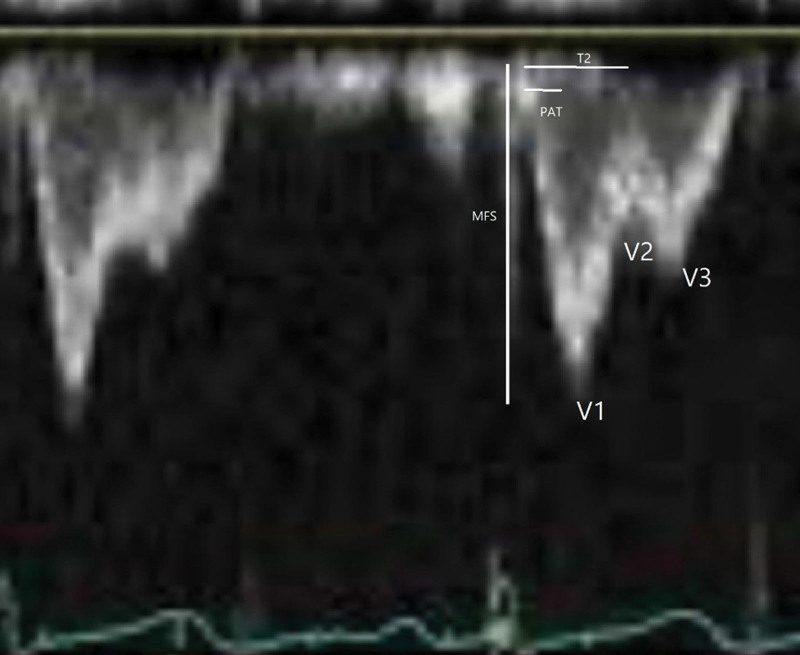
The ratio of MFS to PAT gives us PAS. MFS = maximal frequency shift, PAS = pulmonary artery stiffness, PAT = pulmonary acceleration time, T_2_ = time from onset of pulmonary flow ejection to V_2_, V_1_ = first pulmonary valve pulse Doppler velocity, V_2_ = second pulmonary valve pulse Doppler velocity, V_3_ = third pulmonary valve pulse Doppler velocity.

**Figure 2.**
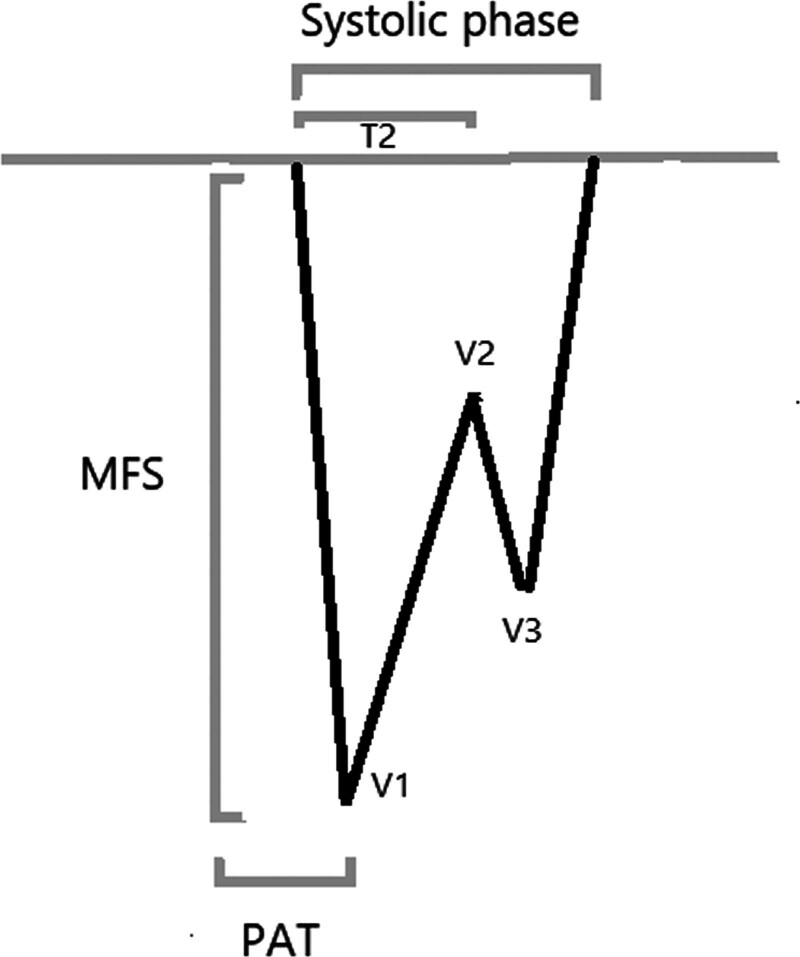
Schematic measurement of echocardiographic evaluation. MFS = maximal frequency shift, PAT = pulmonary acceleration time, T_2_ = time from onset of pulmonary flow ejection to V_2_, V_1_ = first pulmonary valve pulse Doppler velocity, V_2_ = second pulmonary valve pulse Doppler velocity, V_3_ = third pulmonary valve pulse Doppler velocity.

The echocardiographic measurements were performed based on Figure 1. The definition of the measurement parameters is presented in Table 1. Moreover, schematic view of echocardiographic evaluation can be seen in Figure 2.

**Table 1:** Definition of some echocardiographic measurements in patients with PAH.

Measurements	Definition/calculation
PAT (ms)	Time from onset of pulmonary flow ejection to V_1_
V_1_ (ms/s)	First pulmonary valve pulse Doppler velocity
V_2_ (ms/s)	Second pulmonary valve pulse Doppler velocity
V_3_ (ms/s)	Third pulmonary valve pulse Doppler velocity
PAS	MFS/PAT
T_2_ (ms)	Time from onset of pulmonary flow ejection to V_2_
Z_1_	T_2_/V_1_
Z_2_	T_2_/V_2_
Z_3_	(PAT + T_2_/2)/V_1_

MFS = maximal frequency shift, PAS = pulmonary artery stiffness, PAH = pulmonary artery hypertension, PAT = pulmonary acceleration time, T_2_ = time from onset of pulmonary flow ejection to V_2_, V_1_ = first pulmonary valve pulse Doppler velocity, V_2_ = second pulmonary valve pulse Doppler velocity, V_3_ = third pulmonary valve pulse Doppler velocity.

### 2.6. Ethics

Written informed consent was obtained from all patients. This study was approved by the local ethics committee of the Mashhad University of Medical Sciences (Code: IR.MUMS.MEDICAL.REC.1399.437) in compliance with the Declaration of Helsinki.

### 2.7. Statistics

Data were entered in the statistical package for social sciences (SPSS) software (SPSS, Ver. 18.0, 2018; SPSS Inc., Chicago, IL). Qualitative variables were presented as percent and frequency and percentage. Continuous quantitative variables were reported as mean and standard deviation. Correlation between quantitative variables was evaluated by Spearmen or Pearson tests. For categorical variables, Chi-square or Fisher exact tests were used. The relationship between echocardiographic parameters and 6MWT was assessed using linear regression. A *P* < .05 was considered statistically significant.

## 3. Results

We included 50 patients in this study. The mean age of the patients was 41.90 ± 14.73 years old. The majority of the patients were female (37, 74%). The most common cause of pulmonary hypertension was idiopathic (37, 74%).

The mean wedge pressure measured by right heart catheterization was 8.02 ± 3.21 cm Hg. Table 2 shows the demographic, clinical, and general information of the study patients.

**Table 2:** General characteristics of the study patients.

Characteristics	
Age (mean ± SD) (yr)	41.90 ± 14.73
Sex (n, %)	Female (37, 74), male (13, 26)
Etiology of pulmonary hypertension (n, %)	AP window (1, 2)
	ASD (4, 8)
	Idiopathic (37, 74)
	Lupus (1, 2)
	PDA (2, 4)
	PTE (1, 2)
	Scleroderma (1, 2)
	TA (1, 2)
	VSD (2, 4)
BSA (m^2^) (mean ± SD)	1.72 ± 0.15
6MWT (m) (mean ± SD)	400.14 ± 79.53
Smoker (n, %)	12, 50

6MWT = 6-min walking test, AP = aortopulmonary, ASD = atrial septal defect, BSA = body surface area, PDA = patent ductus arteriosus, PTE = pulmonary thromboembolism, SD = standard deviation, TA = truncus arteriosus, VSD = ventricular septal defect.

The mean of PAS measured by echocardiography was 41.2 ± 5.38. Other echocardiographic parameters in the study patients are presented in Table 3.

**Table 3:** Echocardiographic parameters.

Variable	Mean ± SD
E/E’ ratio	7.66 ± 3.83
PAS (kHz/s)	41.2 ± 5.38
TAPSE (cm) (mean ± SD)	1.34 ± 0.23
mPAP (mm Hg) (mean ± SD)	68.95 ± 18.25
PASP (mm Hg) (mean ± SD)	109.53 ± 27.39
FAC	22.40 ± 5.09

E/E’m = ratio of early diastolic trans tricuspid flow velocity to early diastolic peak myocardial velocity, FAC = fractional area change, mPAP = mean pulmonary arterial pressure, PAS = pulmonary artery stiffness, PASP = pulmonary artery systolic pressure, SD = standard deviation, TAPSE = tricuspid annular plan systolic excursion.

As it is shown in Table 4, there was a significant correlation between PAS and pulmonary artery systolic pressure (PASP) (*r* = 0.302, *P* = .041), but there was no significant correlation between PAS and 6MWT, PVR, and tricuspid annular plane systolic excursion (TAPSE) (*P* > .05).

**Table 4:** Correlation between PAS and other factors.

	6MWT	PASP	PVR	FAC	TAPSE
PAS					
Correlation coefficient	0.040	0.302	0.045	−0.042	−0.111
*P* value	.392	.041	.420	.390	.230

6MWT = 6-min walking test, FAC = fractional area change, PAS = pulmonary artery stiffness, PASP = pulmonary artery systolic pressure, PVR = pulmonary vascular resistance, TAPSE = tricuspid annular plan systolic excursion.

**Figure 3.**
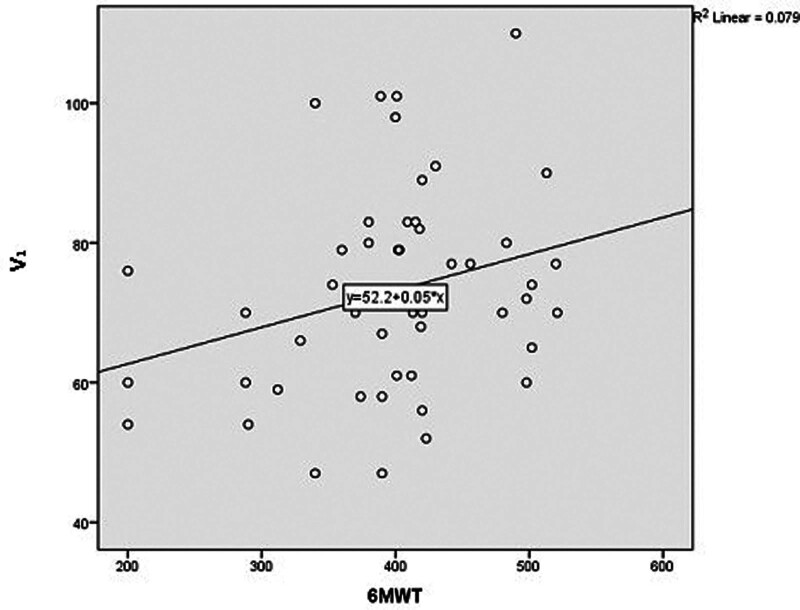
The correlation between V_1_ and 6MWT. 6MWT = 6-min walk test, V_1_ =  first pulmonary valve pulse Doppler velocity.

There was a significant correlation between first pulmonary valve pulse Doppler velocity (V_1_) and 6MWT (*r* = 0.259, *P* = .036) (Fig. 3). There was a significant correlation between PASP and second pulmonary valve pulse Doppler velocity (*r* = −0.461, *P* = .003), time from onset of pulmonary flow ejection to second pulmonary valve pulse Doppler velocity/V_1_ (*r* = −0.311, *P* = .037), and Z_3_ ratio (*r* = −0.346, *P* = .023). There was also a significant correlation between second pulmonary valve pulse Doppler velocity and PVR (*r* = 0.359, *P* = .049). There was no significant correlation between 6MWT with PASP (*r* = 0.067, *P* = .353), PVR (*r* = −0.069, *P* = .381), and PAS (*r* = 0.04, *P* = .392). Multivariate regression analysis showed that PAS and V_1_ could significantly predict functional capacity assessed by 6MWT (Table 5).

**Table 5:** Multivariate logistic regression analysis for 6MWT.

Variable	OR	*P* value	95% confidence interval
PAS	0.738	.008	−349.20 to −60.00
PV 1	0.599	.027	0.54–7.95

6MWT = 6-min walking test, OR = odds ratio, PAS = pulmonary artery stiffness, PV1 = first pulmonary valve pulse Doppler velocity.

## 4. Discussion

The main finding in our study was that V_1_ could significantly predict functional capacity in patients with PAH but PAS was not significantly correlated with 6MWT. We also found some new indices that were correlated with PASP in our study population.

Arterial stiffness is a well-known risk factor for cardiovascular events and the secondary target for pharmacological treatment.^[17]^ Endothelial dysfunction is one of the main factors associated with the development of both PAH^[18,19]^ and systemic hypertension.^[20,21]^ Similar to systemic hypertension and regardless of blood pressure levels, arterial stiffening (demonstrated by reduced compliance and distensibility) emerged as another distinctive feature of cardiovascular risk, which can be invariably observed concurrently with endothelial dysfunction.^[22]^ The pulmonary artery is a low-pressure, high-distensible system that transforms the highly pulsatile RV output into the near steady flow at the capillary level.^[23]^ The distal pulmonary vascular bed is also comprised of highly distensible vessels. Distension and recruitment of this microvascular bed can accommodate large increases in volume such as during exercise. This response is the opposite of that experienced in systemic circulation where exercise induces vasoconstriction in the majority of the vascular tree other than the vessels supplying muscles. This inbuilt reserve means that resting PAPs will rise relatively late in a disease process only when 60% to 70% of the bed is obstructed.^[13]^

PAH is a rare but fatal disease worldwide that can be due to many causes ranging from idiopathic to secondary to many underlying pathologies, including congenital heart diseases. Several prognostic indicators, including New York Heart Association functional class, 6MWT, and echocardiographic and hemodynamic parameters, are used for routine follow-up of the disease. Right heart catheterization is pivotal in the evaluation of patients with PAH. This invasive test is used for both diagnosis and determination of the prognosis of PAH.^[19]^ However, the most widely used method in routine practice is Doppler echocardiography. Right heart catheterization may not be suitable for routine evaluation due to its invasiveness. On the other hand, echocardiography has some limitations in the diagnosis of PAH. For instance, systolic PAP cannot be estimated in echocardiography based on a modified Bernoulli equation in the absence of tricuspid regurgitation. Therefore, it is necessary to develop new noninvasive methods. Studies found a correlation between pulmonary artery elasticity and PASP. Studies have demonstrated that PAS parameters, which are derived from cardiac magnetic resonance (such as ventricular mass index, interventricular septal configuration, and average pulmonary artery velocity derived by cardiac magnetic resonance), can be used in the determination of PAH.^[15]^ In this investigation, we decided to assess the relationship between PAS and 6MWT in patients with PAH. To the best of our knowledge, no similar study has yet been conducted on this topic. Nevertheless, the findings of our study were in line with the findings of some investigations. Mahfouz^[11]^ assessed the impact of PAS by echocardiography on the long-term RV function and tricuspid regurgitation changes after percutaneous balloon mitral valvuloplasty. They showed that patients with mitral stenosis had significantly higher PAS compared with control subjects. They also showed that PAS was significantly low in patients with progressive RV function improvement and regression of tricuspid regurgitation. They showed that PAS was significantly correlated with the degree of PASP, TAPSE, and ratio of early diastolic trans tricuspid flow velocity to early diastolic peak myocardial velocity ratio.^[11]^ We did not find any significant relationship between PAS and PASP, TAPSE, and ratio of early diastolic trans tricuspid flow velocity to early diastolic peak myocardial velocity ratio.

It is believed that vasoconstriction develops in the early stages of PAH and that an increase in vasoconstriction leads to endothelial dysfunction. Elastic and collagen fibers are important components of the arterial wall that determine the extensibility of the great arteries.^[24]^ Pulmonary artery and vascular bed changes occur before the development of overt PAH. Therefore, a remarkable increase in PAS might be a predictor for the onset of changes in the pulmonary vascular bed.^[25]^ Previous studies have shown that emphasis on the increase in PASP may result in failure to diagnose these early changes. Our study demonstrated a significant correlation between PAS and PASP, which can be useful for early diagnosis of PAH especially in patients with predisposing underlying diseases. Weir-McCall et al^[13]^ reviewed the role of PAS in chronic obstructive pulmonary disease (COPD) patients. They showed that PAS increases with the severity of COPD, and correlates well with the presence of exercise-induced pulmonary hypertension. They reported a curvilinear relationship exists between PA distensibility and mPAP and PVR with a marked loss of distensibility followed by a rapid rise in mPAP and PVR that occurs with the resultant RV failure. Weir-McCall et al^[13]^ concluded that PAS can be a promising biomarker for early detection of pulmonary vascular disease, and can play a role in RV failure in COPD. Ozkececi et al^[26]^ evaluated PAS and cardiac function in 60 newly diagnosed patients with obstructive sleep apnea syndrome (OSAS) and 30 healthy controls. They analyzed the relationship between OSAS severity and PAS. Positive correlations were observed between the apnea-hypopnea index and total oxygen desaturation with PAS and mean PAP. They found that PAS and mPAP were increased in patients with OSAS and that the pulmonary vascular bed may be affected by the diurnal and nocturnal fluctuation of PAP.^[26]^ Obstructive sleep apnea appears to have an independent effect on arterial stiffness, which may be one of the mechanisms explaining sleep apnea-associated cardiovascular risk.^[27]^

Some studies have demonstrated that increased PAS is a predictor of decreased functional capacity. Kang et al^[6]^ showed that the noninvasive cardiac magnetic resonance imaging-derived PA distensibility index correlates with PA stiffness and can predict functional capacity with 6MWT in patients with PAH. The 6MWT is an important functional test in the assessment of functional capacity in PAH patients. It is an important parameter for clinical surveillance of disease progression and treatment effects. In this study, there was no significant correlation between PAS and 6MWT. We used novel parameters to assess the relationship between echocardiography and 6MWT. V_1_ as defined in the Methods section, was found to have a significant positive correlation with 6MWT. We also demonstrated in multivariate regression analysis that PAS and V_1_ significantly predicted the 6MWT. 6MWT is a primary endpoint of many clinical investigations related to PAH. Therefore, these variables can be used for future clinical studies in the evaluation of PAH severity and response to treatment.

PA distensibility index using noninvasive methods such as echocardiography or computed tomography was introduced to easily measure PA stiffness. These methods have some limitations, including reproducibility and variability and lack of data to compare with invasive hemodynamic parameters.^[28,29]^ This study has some limitations; our sample size was small and the nature of this study was descriptive. So, the interpretation of the findings should be addressed with caution.

## 5. Conclusion

To the best of our knowledge, this study was the first study that assessed the relationship between functional capacity and PAS and V_1_ in PAH patients with any underlying predisposing factors. These parameters are simple, noninvasive, available, and easy-to-measure tools for the evaluation of pulmonary vascular beds and diagnosis of presymptomatic clinical status in patients with PAH. We have shown that these new echocardiographic parameters can have diagnostic value for pulmonary hypertension. Large-scale multicentric studies on this issue can be helpful to identify the power of these factors.

## Acknowledgments

We all thank nursed of Ghaem Hospital helped us in different stages of patient evaluations.

## Author contributions

**Conceptualization:** Leila Bigdelu, Sara Afshar.

**Data curation:** Leila Bigdelu, Hedieh Alimi, Zahra Abbasi Shaye, Sara Afshar.

**Formal analysis:** Leila Bigdelu, Zahra Abbasi Shaye, Sara Afshar.

**Investigation:** Leila Bigdelu, Sara Afshar.

**Methodology:** Leila Bigdelu, Faeze Keihanian, Sara Afshar.

**Project administration:** Leila Bigdelu.

**Resources:** Afsoon Fazlinezhad, Fereshteh Ghaderi, Hoorak Poorzand, Hedieh Alimi, Farveh Vakilian, Faeze Keihanian.

**Supervision:** Leila Bigdelu, Afsoon Fazlinezhad, Hoorak Poorzand, Farveh Vakilian.

**Validation:** Hedieh Alimi, Afsoon Fazlinezhad, Fereshteh Ghaderi, Hoorak Poorzand, Zahra Abbasi Shaye.

**Visualization:** Hedieh Alimi, Fereshteh Ghaderi, Hoorak Poorzand.

**Writing – original draft:** Faeze Keihanian, Sara Afshar.

**Writing – review & editing:** Faeze Keihanian, Sara Afshar.
